# Direct loop-mediated isothermal amplification assay for on-site detection of *Staphylococcus aureus*

**DOI:** 10.1093/femsle/fny092

**Published:** 2018-04-10

**Authors:** Xiaolan Tian, Junli Feng, Yi Wang

**Affiliations:** 1 Institute of Seafood, Zhejiang Gongshang University, Hangzhou 310012, PR China; 2 Key Lab of aquatic Products Processing of Zhejiang Province, Hangzhou 310012, PR China

**Keywords:** food-borne pathogen; *Staphylococcus aureus*, direct LAMP, on-site detection

## Abstract

*Staphylococcus aureus* (*S*. *aureus*) is a major human pathogen that may produce a variety of toxins and cause staphylococcal food poisoning. In the present study, a direct loop-mediated isothermal amplification (LAMP) assay was developed and validated to detect *S. aureus* in food samples. Without prior cultural enrichment and DNA extraction steps, bacterial DNA was released by heating at 100°C for 5 min and directly subjected to LAMP assay. Using a set of LAMP primers recognizing six distinct regions of *nuc* gene, the developed direct LAMP assay was highly specific, and the analytical sensitivity was determined to be 7.6 × 10^2^ CFU/mL. Moreover, with the pre-mixed LAMP reagents stored at –20°C, the entire assay should be finished within 40 min. These features greatly simplified the operating procedure and made the direct LAMP a powerful tool for rapid and on-site detection of *S. aureus* in food samples.

## Teaser

The direct LAMP is a powerful tool for rapid and on-site detection of *S. aureus* in food samples.

## INTRODUCTION

*Staphylococcus aureus* (*S. aureus*) is an extremely common pathogenic bacterium that can be found in a number of different food varieties, including mixed foods (pasta dishes, salads), meat and meat products, egg and egg products, vegetables, baked goods and cheeses (Zeleny *et al.*^2015^). Due to its multi-drag resistance and virulence, *S. aureus* has become a major concern to public health and food safety in many countries (Sheet *et al.*^2016^; Shin *et al.*^2016^; Jans *et al.*^2017^; Lidiane *et al.*^2017^; Miao *et al.*^2017^; Miao *et al.*^2017^). According to the reports from Chinese Center for Disease Control and Prevention, there are 240 000 illnesses annually in China with 1000 hospitalizations and six deaths induced by staphylococcal food poisoning (Scallan *et al.*^2011^). Therefore, *S. aureus* detection is one of the routine programs for food safety testing in China (Chinese National food safety standard GB 4789.10-2010).

Nowadays, culture-based method is still used as a routine method for *S. aureus* detection. This conventional method involves enrichment and enumeration in liquid media, subsequent recovery and isolation of colonies on selective culture broth and further confirmation assays (Chinese National food safety standard GB 4789.10-2010). Generally, it requires a lengthy time span of 3–5 d. Molecular biology techniques such as polymerase chain reaction (PCR), real-time PCR and DNA hybridization have been developed and become popular for pathogen identification (Sudhaharan *et al.*^2015^). Although these technologies can detect low number of bacterial cells, they usually need several hours. Moreover, such techniques required prior cultural enrichment and bacterial DNA isolation, preparation of enzyme reaction mixtures and expensive equipment (Kei *et al.*^2014^). Therefore, most of these techniques are not suitable for on-site detection. Nevertheless, loop-mediated isothermal amplification (LAMP) is a simple, cost-effective and rapid method that is often used as an alternative to PCR-based methodologies in pathogen detection (Ihira *et al.*^2004^; Kurosaki *et al.*^2007^; Kanitkar *et al.*^2017^; Lin *et al.*^2017^; Yan *et al.*^2017^). Nowadays, LAMP has been applied to detection many pathogens such as *Salmonella*, *Escherichia coli*, *Pseudomonas aeruginosa*, *Vibrio parahaemolyticus* and *Listeria monocytogenes* (Xu *et al.*^2010^; Zhao *et al.*2010a,b; Wang *et al.*^2011^; Zhao *et al.*^2011^; Wang *et al.*^2012^; Liu *et al.*^2016^, ^2017^). Besides these, LAMP assays were also demonstrated to be useful and powerful tools for examination of various *Staphylococcus* strains (Misawa *et al.*^2007^; Xu *et al.*^2012^; Wang *et al.*^2015^). Most of these studies were based on detection the genetic elements of staphylococcal chromosomal cassette *mec* (such as *mecA*, *orfX* and *femA*), which has been reported to play a core role in the antimicrobial resistance characteristics, evolution and molecular epidemiology of staphylococcal diseases (Xu *et al.*^2011^; Liu *et al.*^2016^). Due to its simplicity and easiness of performance without any sophisticated instrumentation, LAMP can be easily adapted to any microbiology laboratory and on-site detection as well (Sudhaharan *et al.*^2015^).

Like other kinds of molecular methods, bacterial DNA extraction and purification are the primary steps for LAMP assay, which have become a bottleneck for rapid detection (Yan *et al.*^2017^). Actually, an easy, effective and low-cost DNA isolation protocol will greatly accelerate the detection process and broaden its practical application. Several studies have reported the successful production of DNA template (for PCR or LAMP assay) by simply boiling the specimens (Koizumi *et al.*^2012^; Wang *et al.*^2012^; Kei *et al.*^2014^; Wozniakowski and Samorek-Salamonowicz 2014; Wozniakowski *et al.*^2015^; Imai *et al.*^2018^). Therefore, we decided to take advantage of LAMP using boiled *S. aureus* cultures as DNA templates in this study. Moreover, the LAMP reagents were pre-mixed and stored at −20°C. With such preparation, the method can deliver a ‘sample-to-result’ time of approximately 30 min. Besides these, the results of LAMP reaction were visualized by naked eyes. These characters demonstrated the feasibility of direct LAMP to be used as a rapid and effective on-site method for *S. aureus* detection.

## MATERIALS AND METHODS

### Bacterial strains

*Staphylococcus aureus* ATCC 6538, *Listeria monocytogenes* ATCC 19112, *Escherichia coli* ATCC 25922, *Sligella flexneri* ATCC 12022 and *Salmonella enteritidis* ATCC 15611, *Vibrio parahaemolyticus* 17802, *Bacillus cereus* ATCC 11778 and *Clostridium perfringens* ATCC 13124 were purchased from the American Type Culture Collection (ATCC). All of the bacterial strains were cultured in tryptone soy broth (TSB) or lysogeny broth (LB) broth/agar plates at 37°C, with media purchased from Beijing Land Bridge Technology Co. Ltd (Beijing, China). These bacteria were harvested from fresh cultures, and were decimal diluted by double-distilled H_2_O (ddH_2_O).

### Preparation of *S. aureus* genomic DNA and template of LAMP assay

After *S. aureus* ATCC 6538 was cultivated at 37°C for 24 h, its concentration was precisely determined by plate-counting technique and expressed as colony forming unit per mL of bacterial culture (CFU/mL). Then, bacterial DNA was isolated either by a ‘Bacterial Genomic DNA Extraction kit’ (Takara Biotechnology, Dalian, China), according to the manufacturer's instructions, or by heating the cell at 100°C for 5 min and immediately chilling on ice. One milliliter fresh cultured *S. aureus* (about 10^6^–10^8^ CFU/mL) was used for each extraction. All of the extractions were carried out in triplicate, and stored at −20°C until further used.

### Primer design

The *nuc* gene of *S. aureus* ATCC 6538 was downloaded from NCBI database (http://www.ncbi.nlm.nih.gov/, accession number EF253320). Based on the sequence information and previous report (Sudhaharan *et al.*^2015^), a set of LAMP primers targeting *nuc* gene was chosen. At the same time, the forward and reverse PCR primers of *nuc* gene were also designed by Primer Premier 5.0 (PREMIER Biosoft International, Palo Alto, CA, USA). These primer sequences were tested against the Basic Local Alignment Search Tool (National Center of Biotechnology Information (NCBI), http://blast.ncbi.nlm.nih.gov/Blast.cgi) to ensure specificity. All of the primers were synthesized by Sangon Biotech (Shanghai, China), and listed in Table 1.

**Table 1.** Details of the LAMP and PCR primers used in this study.

Primer	Primer sequence (5'→ 3')	Reference
LAMP assay		
*nuc*-F3	TCGCTTGCTATGATTGTGG	(Miao *et al.*^2017^)
*nuc*-B3	ACATACGCCAATGTTCTACC	
*nuc*-FIP	GTACAGTTTCATGATTCGTCCCGCCATCATTATTGTAGGTGT	
*nuc*-BIP	TGTTCAAAGAGTTGTGGATGGTGTACAGGCGTATTCGGTT	
*nuc*-FLP	TTGAAAGGACCCGTATGATTCA	
*nuc*-BLP	GATACGCCAGAAACGGTGA	
PCR assay		
*nuc*-F	TGTTACTTATAGGGATGGCTATCAG	Designed in this study
*nuc*-R	AGTTAACACTAAGCAACTAGTAGCG	

### LAMP reaction

The LAMP reaction was carried out using the DNA thermostatic amplification kit (Guangzhou Deaou Bio-technology Co., Ltd, Guangdong, China) following the manufacturer's instructions. The visual LAMP detection was performed in a 10 μL reaction mixture containing 5 μL Reaction Buffer (2×), 0.2 μM each of F3/B3, 1.6 μM each of FIP/BIP, 0.4 μM each of FLP/BLP, 3.2 U *Bst* 2.0 WarmStart DNA polymerase, 0.5 μL *S. aureus* DNA template and 2.26 μL ddH_2_O. As described in our previous works, 10 μL mineral oil was added to the top of reaction mixture, and 0.4 μL dye (SYBR Green® I, Guangzhou Deaou) was added on the inner wall of tube cap of each reaction (Liu *et al.*^2016^; Ye *et al.*^2016^, ^2017^). Reaction tube was inoculated at 63°C for 20–40 min, and thereafter the reaction solution was mixed with SYBR Green® I by shaking. All reactions were run in triplicate, and the negative controls were performed using sterile water instead of bacterial DNA template. The reaction was considered as positive if its color turned from orange to green under natural light, whereas for negative reactions the solution retained the original orange color.

The real-time fluorescence LAMP detection was carried out on the Mini Opticon Real-time PCR System (Bio-Rad Laboratories, Hercules, CA, USA), with reaction components similar to visual LAMP. However, instead of the SYBR Green® I and mineral oil, the SYTO® 9 Green (fluorescent nucleic acid stain, Invitrogen Corp., Carlsbad, CA) was added into the reaction mixture. Like real-time PCR, fluorescence signals of the reaction were collected at 1-min intervals, and a typical sigmoid shaped fluorescence curve was produced. The threshold line of this fluorescence curve was set using the equation:

\begin{equation*}
{\rm{Threshold}} = 1{\rm{0}} \times {\rm{S}}{{\rm{D}}_{{\rm{(1st - 10th)}}}}{\rm{;}}
\end{equation*}

where SD_(1st–10th)_ is the standard deviation of fluorescence signals from first to tenth min. And the time point when fluorescence curve reached threshold line was called as ‘detection time.’

### Determination the specificity and sensitivity of LAMP assay

In order to determine the specificity of LAMP assay, the visual detection were carried out under the conditions described above, using DNA templates extracted from *S. aureus*, *L. monocytogenes*, *E. coli*, *S. flexneri*, *S. enteritidis*, *V. parahaemolyticus*, *B. cereus* and *C. perfringens*. The sensitivity of LAMP assay was assessed using DNA samples extracted from *S. aureus* of various concentrations. Briefly, the *S. aureus* cells were serially diluted with ddH_2_O, to get final concentrations ranging from 7.6 to 7.6 × 10^6^ CFU/mL. Then, 1 mL of each dilution was used for DNA extraction, and subsequently subjected to LAMP assay as described above. The limit of detection (LOD) was expressed as the lowest concentration of *S. aureus* (CFU/mL) that gave a positive result in all of three individual replicates.

### Stability of pre-mixed LAMP reagents and its application in real food samples

In order to reduce the time and complexity of detection, the reagents for visual LAMP reaction were pre-mixed (including 1× reaction buffer, 0.2 μM of each F3/B3, 1.6 μM of each FIP/BIP, 0.4 μM of each LF/LB, 0.32 U/μL of *Bst* 2.0 WarmStart DNA polymerase and mineral oil), and named as reagent A (Guangzhou Deaou). Reagent B was SYBR Green® I, which served as indicator (Guangzhou Deaou). Reagents A and B were stored at 4°C and −20°C, and tested periodically for up to 4-weeks. And the results were compared with that obtained by the conventional LAMP assay.

Moreover, the feasibility of developed LAMP with pre-mixed LAMP reagents was analyzed. For this purpose, 18 real food samples collected from local markets were subjected to *S. aureus* detection. The detection was performed as follows: 1 g of each sample was weight into a tube and mixed with 9 mL of sterilized water. The mixture was stand for 2 min, and 1 mL supernatant was used for DNA extraction by heating at 100°C for 5 min. Subsequently, these DNA samples were subjected to both LAMP and conventional PCR assays. The results of LAMP reaction were visualized as described earlier, and the PCR products were judged by gel electrophoresis. Each sample was tested twice to ensure reproducibility.

## RESULTS

### Reaction time of the direct LAMP assay

**Figure 1.**
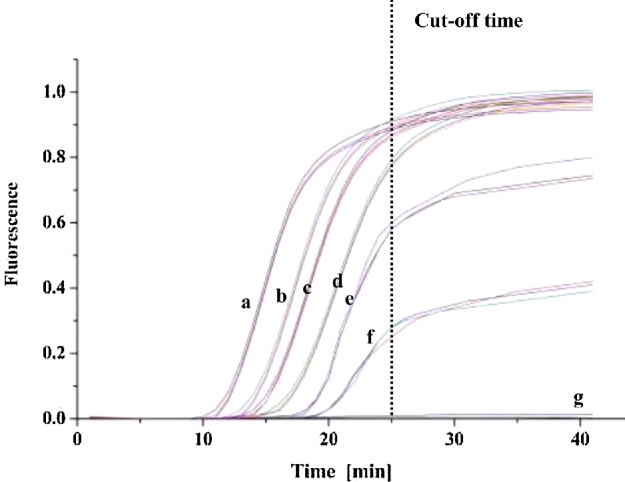
Determination the reaction time of direct LAMP assay based on fluorescence signals. The real-time fluorescence LAMP was carried out using DNAs extracted from *S. aureus* of 7.6 × 10^6^ CFU/mL (**a**), 7.6 × 10^5^ CFU/mL (**b**), 7.6 × 10^4^ CFU/mL (**c**), 7.6 × 10^3^ CFU/mL (**d**), 7.6 × 10^2^ CFU/mL (**e**), 7.6 × 10^1^ CFU/mL (**f**), 7.6 CFU/mL and negative controls (**g**). Cut-off time of the detection was set as 25 min (vertical dash line).

In the LAMP assay, the reaction temperature and optimum concentration of each component were determined based on pre-experiment and our previous works (as described in materials and methods; Liu *et al.*^2016^; Ye *et al.*^2016^, ^2017^). Under such conditions, the RealAmp reaction was performed to determine the optical amplification time, by *S. aureus* cells with concentrations ranged from 7.6–7.6 × 10^6^ CFU/mL. Using DNA extracted from these diluted *Staphylococcus aureus* by commercial kit, the LAMP reactions with *S. aureus* cells higher than 7.6 × 10^1^ CFU/mL were successfully amplified within 25 min (Fig. 1). Thus, cut-off time of this method was set as 25 min as the vertical dash line shown in Fig. 1, and amplifications with detection time more than 25 min would be considered as negative reactions.

### Specificity of the *S. aureus* direct LAMP assay

**Figure 2.**
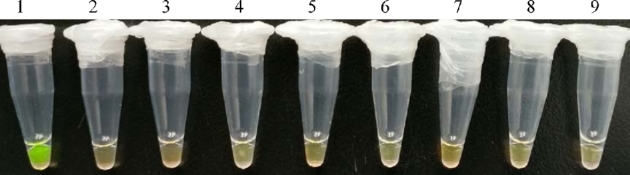
Specificity test of *S. aureus* by direct LAMP assay. Visual LAMP was carried out using DNA isolated from *S. aureus* (Lane 1), *L. monocytogenes* (Lane 2), *E. coli* (Lane 3), *S. flexneri* (Lane 4), *S. enteritidis* (Lane 5), *V. parahaemolyticus* (Lane 6), *B. cereus* (Lane 7), *C. perfringens* (Lane 8) and negative control (Lane 9). Each assay was carried out in triplicate.

Specificity of the assay was evaluated by testing the amplification performance of the developed direct LAMP with DNA isolated from *S. aureus* and seven other bacterial species. As shown in Fig. 2, after incubation at 63°C for 25 min, only the solution color of reaction including *S. aureus* DNA turned from orange to green (line 1) in visual LAMP assay. No cross-reactivity was observed for other bacterial species. Thereafter, the LAMP assay was highly specific, which might result from the specific targeting of *nuc* gene by LAMP primers set.

### Sensitivity and Detection limit of *S. aureus* in different LAMP assays

**Figure 3.**
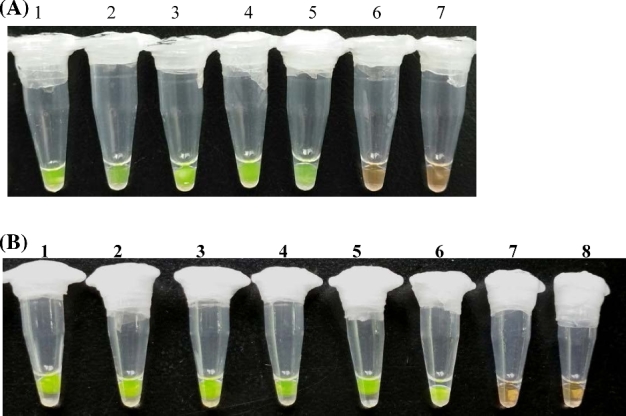
Sensitivity test of *S. aureus* by different LAMP assays. Visual LAMP detection of *S. aureus* via SYBR Green^®^ I staining. The LAMP assay was performed using DNA extracted by boiling the samples (**A**), and commercial kit (**B**). Lane 1–7, LAMP assays using DNA templates extracted from *S. aureus* cell of 7.6 × 10^6^ CFU/mL, 7.6 × 10^5^ CFU/mL, 7.6 × 10^4^ CFU/mL, 7.6 × 10^3^ CFU/mL, 7.6 × 10^2^ CFU/mL, 7.6 × 10^1^ CFU/mL, and 7.6 × 10^0^ CFU/mL. Lane 8, negative control, in which no DNA template was added in the reaction mixture. Each assay was carried out in triplicate.

Sensitivity of the ‘direct LAMP’ was analyzed using fresh cultured *S. aureus* cells. The cells were 10-fold serially diluted, and bacterial DNA was obtained by boiling the samples. As the results shown in Fig. 3a, the LOD was determined to be 7.6 × 10^2^ CFU/mL of *S. aureus* cells.

In parallel, the sensitivity of LAMP was also determined with serially diluted DNA samples of *S. aureus*. Using DNA samples extracted by commercial kit, the LOD was determined to be 7.6 × 10^1^ CFU/mL in Visual-LAMP assays (Fig. 3b).

### Stability of the pre-mixed LAMP reagents

**Figure 4.**
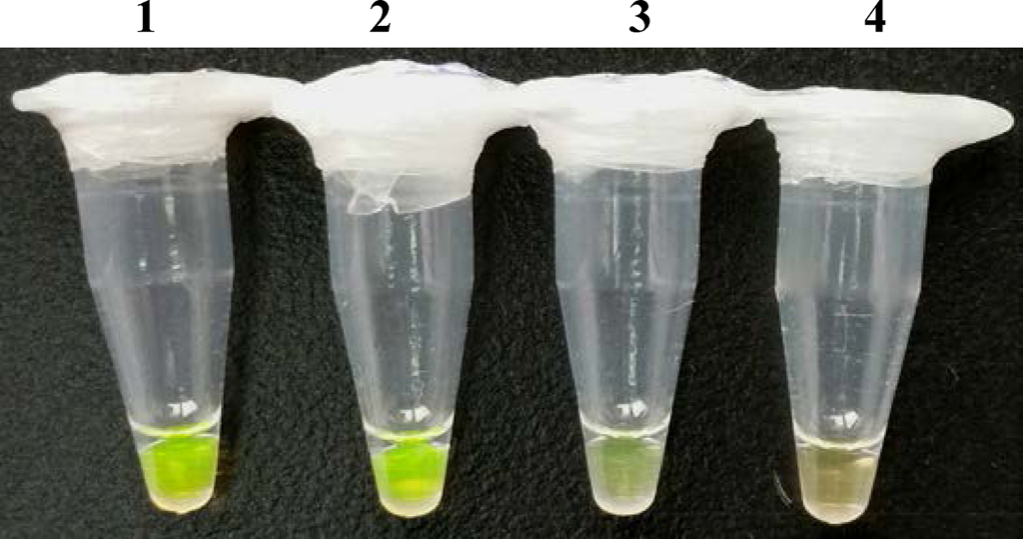
Stability test of the pre-mixed LAMP reagents. Visual detection of *S. aureus* using conventional LAMP reagents (Lane 1), pre-mixed LAMP reagents stored at –20°C for 24 h (Lane 2), –4°C for 24 h (Lane 3) and negative control (Lane 4).

After the reagent A and B were stored at 4°C and −20°C for 24 h, they were used to detect *S. aureus* by direct LAMP. Compared with LAMP assay using reagents stored at −20°C, the reaction color was obviously faded using reagents stored at 4°C (as shown in Fig. 4, Lane 2 vs Lane 3). Therefore, these results indicated that 4°C was unsuitable for the storage of the pre-mixed reagents.

Subsequently, the stability of pre-mixed LAMP reagents stored at −20°C was further analyzed. After stored for 7, 14, 21 and 28 days, the LAMP reactions were carried out and recorded. No color differences were observed among these reactions, which indicated that pre-mixed LAMP reagents were stable under −20°C.

### Evaluation of the direct LAMP assay using pre-mixed reagents in real food samples

**Figure 5.**
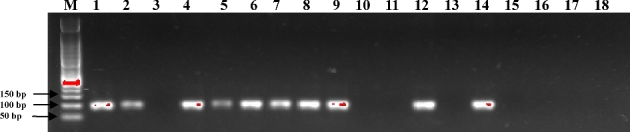
Detection of *S. aureus* in real food samples by conventional PCR assays. Lane M: 50 bp DNA marker; Lane 1–3, smoked chicken samples; Lane 4–6, duck egg samples; Lane 7–8, minced pork samples; Lane 9–10, pork slice samples; Lane 11, cooked beef sample; Lane 12, squid (raw) sample; Lane 13, shrimp (raw) sample; Lane 14, surimi sample; Lane 15, roast fish sample; Lane 16, smoked salmon sample; Lane 17–18, dumpling (raw) samples. Each assay was carried out in triplicate.

To evaluate the feasibility of the developed direct LAMP for visual detection of *S. aureus*, 18 real food samples were analyzed using the pre-mixed LAMP reagents stored at −20°C. As the results shown in Table 2, sample 1, 2, 4, 5, 6, 7, 8, 9, 12, 14 were determined to be *S. aureus* positive by both methods. These results were also confirmed by conventional PCR assays (Fig. 5). The consistent results of LAMP and PCR methods indicated the practicality of using the developed direct LAMP in complicated food samples.

**Table 2.** Detection of *S. aureus* in 18 real food samples by direct LAMP.

Sample	Product description	Visual LAMP assays	PCR
1	Smoked chicken-1	+	+
2	Smoked chicken-2	−	+
3	Smoked chicken-3	−	−
4	Duck egg-1 (raw)	+	+
5	Duck egg-2 (raw)	+	+
6	Duck egg-3 (raw)	+	+
7	Minced pork-1	+	+
8	Minced pork-2	+	+
9	Pork slice-1 (cooked)	+	+
10	Pork slice-2 (cooked)	−	−
11	Cooked beef	−	−
12	Squid (raw)	+	+
13	Shrimp (raw)	−	−
14	Surimi	+	+
15	Roast fish	−	−
16	Smoked salmon	−	−
17	Dumpling-1 (raw)	−	−
18	Dumpling-2 (raw)	−	−

+, positive; −, negative.

## DISCUSSION

*Staphylococcus aureus* is one of the most frequent causative agents of food-borne disease. Many studies have reported the detection of *S. aureus* based on the *nuc* gene, as which is specific for *S. aureus* (Costa, Kay and Palladino 2005; Delibato *et al.*^2005^; Pinto, Chenoll and Aznar 2005; Suhaili *et al.*^2009^; Cattoir *et al.*^2011^; Montazeria *et al.*^2014^; Panda *et al.*^2014^; Montazeria *et al.*^2015^). In this study, the LAMP primers set was also designed based on sequence of *nuc* gene. With inner and outer primers recognizing six distinct regions, the developed LAMP was highly specific (shown in Fig. 2).

In the case of molecular detection of pathogen, it takes at least 1 h for bacterial DNA extraction and purification. To reduce the cost and reaction time, a quick and simple template isolation procedure is in urgent need of LAMP reaction, especially for the on-site detection. It has been reported that plenty of bacterial cells would be leaked and a large amount of DNA would be released after heating at 94°C for 10 min (Lin *et al.*^2017^). According to the results shown in Fig. 3, although the release of bacterial DNA due to cell breakage is 10-fold less efficient compared with DNA extraction using commercial kit, the established direct LAMP was sufficient to yield positive results for *S. aureus* of 7.6 × 10^2^ CFU/mL. Moreover, omission of the DNA extraction procedure would obviously simplify the protocol and shorten the detection time. Based on the Chinese standard GB 29921-2013, the restriction limits of *S. aureus* load in food samples are 10^2^–10^4^ CFU/g. Therefore, the developed direct LAMP has sufficiently sensitivity, and can be used as a fast and easy-to-perform diagnostic procedure for early *S. aureus* detection.

It should be highlighted that the detection time for *S. aureus* by the developed direct LAMP is greatly decreased. As eliminating the prior cultural enrichment and DNA isolation steps, the hands-on time per specimen can be finished within 40 min. The detection time was further reduced as the components of LAMP assay does not need to be pipetted one by one. And this could greatly avoid cross-contamination caused by aerosol as well (Liu *et al.*^2016^; Ye *et al.*^2016^, ^2017^). Compared with the analysis time of at least 3 h by conventional PCR and 2 h by conventional LAMP assays, the time-to-results of direct LAMP is reduced by 4.5 and 3 times, respectively. Although it is less sensitive, these characters makes the direct LAMP has promising potential as a valuable tool for rapid detection of *S. aureus* in food samples, and more appropriate for on-site testing.

The main limitation of this study was the small sample size collected for practical analysis. Further studies with more extensive food samples are needed to determine the feasibility of direct LAMP for *S. aureus* detection. However, the detection accuracy of developed direct LAMP assay was 100% when compared with the conventional PCR method, representing its high reliability for real food samples.

## CONCLUSION

In conclusion, the developed direct LAMP assay is a rapid detection method for identifying *Staphylococcus aureus* with high specificity. In this assay, the DNA isolation procedure was eliminated by heating the specimens at 100°C for 5 min, and the LAMP components does not need to be pipetted one by one when the pre-mixed reagents were used. Under such conditions, the direct viral LAMP assay was reliable, and showed a sensitivity of 7.6 × 10^2^ CFU/ml. The entire detection can be finished within 40 min. These characters make it time- and cost-effective, and more appropriate for rapid on-site detection. Therefore, the method developed in this study could be utilized as an efficient detection tool for early and on-site detection of *S. aureus* in food samples.
